# A case report of cholinesterase inhibitor poisoning: cholinesterase activities and analytical methods for diagnosis and clinical decision making

**DOI:** 10.1007/s00204-020-02741-2

**Published:** 2020-04-17

**Authors:** N. Amend, J. Langgartner, M. Siegert, T. Kranawetvogl, M. Koller, H. John, C. Pflügler, C. Mögele-Schmid, F. Worek, H. Thiermann, T. Wille

**Affiliations:** 1 Bundeswehr Institute of Pharmacology and Toxicology, Neuherbergstraße 11, 80937 München, Germany; 2 Klinikum Landshut, Medical Clinic II, Robert-Koch-Straße 1, 84034 Landshut, Germany

**Keywords:** Organophosphate, Carbamate, Pesticide, Acetylcholinesterase, Oxime

## Abstract

Suicidal ingestion of organophosphorus (OP) or carbamate (CM) compounds challenges health care systems worldwide, particularly in Southeast Asia. The diagnosis and treatment of OP or CM poisoning is traditionally based on the clinical appearance of the typical cholinergic toxidrome, e.g. miosis, salivation and bradycardia. Yet, clinical signs might be inconclusive or even misleading. A current case report highlights the importance of enzymatic assays to provide rapid information and support clinicians in diagnosis and rational clinical decision making. Furthermore, the differentiation between OP and CM poisoning seems important, as an oxime therapy will most probably not provide benefit in CM poisoning, but—as every pharmaceutical product—it might result in adverse effects. The early identification of the causing agent and the amount taken up in the body are helpful in planning of the therapeutic regimen including experimental strategies, e.g. the use of human blood products to facilitate scavenging of the toxic agent. Furthermore, the analysis of biotransformation products and antidote levels provides additional insights into the pathophysiology of OP or CM poisoning. In conclusion, cholinesterase activities and modern analytical methods help to provide a more effective treatment and a thorough understanding of individual cases of OP or CM poisoning.

## Introduction

Deliberate intake of large amounts of organophosphorus (OP) or carbamate (CM) compounds in suicidal intention poses a major problem to the public health sector, particularly in Southeast Asia (Mew et al. 2017). After resorption and distribution, OP and CM bind to the active site serine of the pivotal enzyme acetylcholinesterase (AChE), thereby rendering the enzyme in an inactive form-an irreversible reaction in case of various OP and a temporary reversible one in CM poisoning (Marrs 1993). Subsequent failure in hydrolysis of the neurotransmitter acetylcholine (ACh) results in an endogenous ACh overflow for both types of compounds. Cholinergic overstimulation at muscarinic and nicotinic synapses in combination with disturbance in the central nervous system may ultimately result in respiratory failure, thereby requiring intensive-care treatment (Grob 1956; Kwong 2002; Ciottone 2018; Hulse et al. 2019).

In addition to life-saving care, including artificial ventilation, sedation and cardiovascular stabilization, the current standard of antidotal therapy consists of administration of the competitive antagonist of ACh at the muscarinic receptor, atropine until muscarinic signs cease [absence of miosis and salivation, dry axilla, clear lung, heart rate > 80 beats per minute (bpm)] (Eddleston et al. 2008). Oximes are administered to reactivate AChE if the conjugate of inhibited OP–AChE is per se reactivatable and not yet “aged”, i.e. before a dealkylation reaction results in stabilization of the OP–AChE conjugate (Worek et al. 2016c). In so-called “mega-dose poisoning”, the extremely high OP-body load overwhelms the therapeutic efficacy of oximes and no net reactivation can be determined, although the respective OP–AChE conjugate is reactivatable by a particular oxime. However, an administered oxime enhances the elimination of the OP (pseudocatalytic scavenging) and prolongs the aging half-life by repetitive cycles of reactivation and inhibition; therefore, benefit may be provided in the long run (Thiermann et al. 1997).

**Fig. 1:**
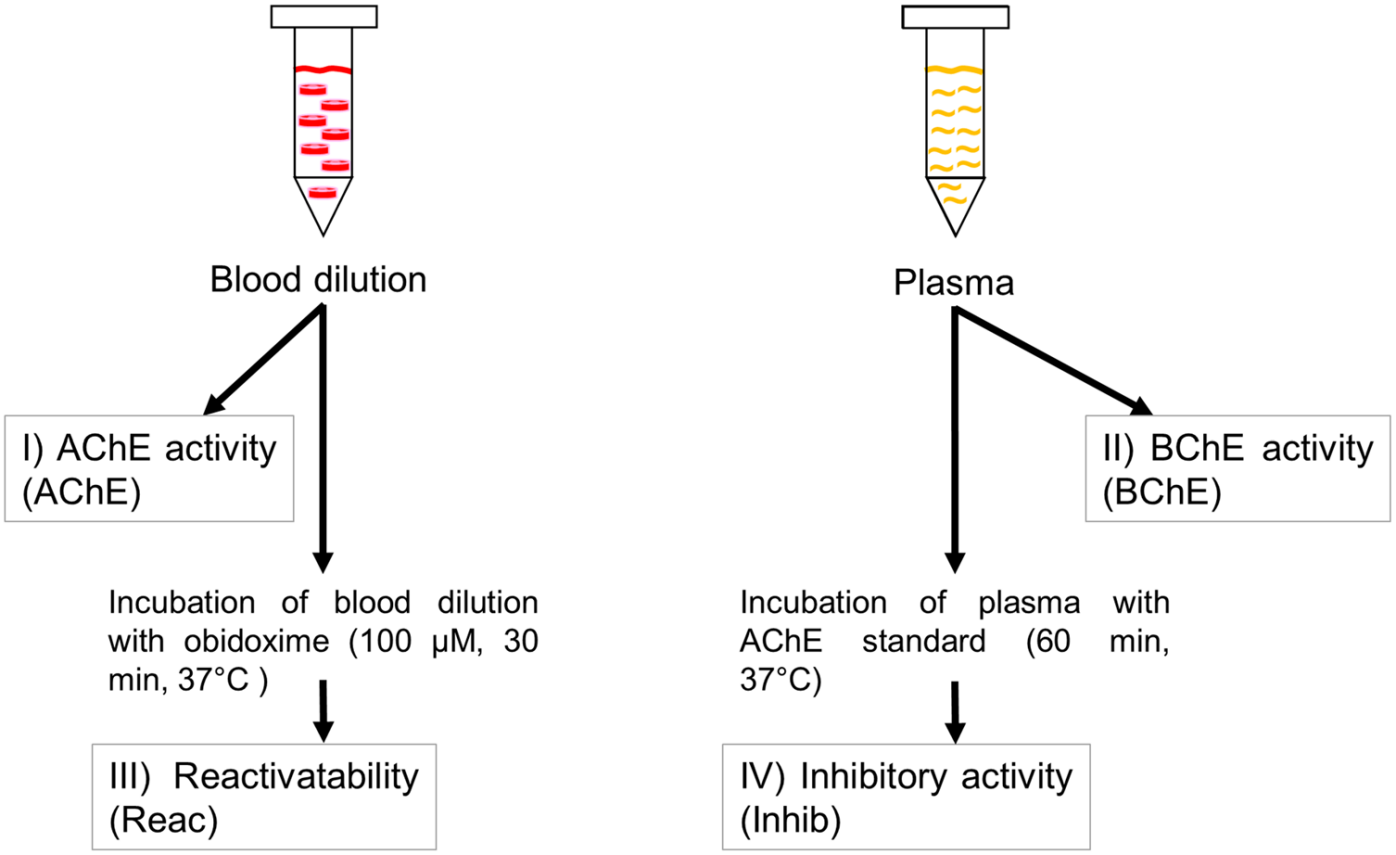
The concept of the cholinesterase status. The concept of the cholinesterase status comprises four independent analyses which corroborate important clinical decisions to facilitate a comprehensive monitoring of the patient’s course of poisoning and necessity of oxime therapy

In contrast to OP poisoning, the effect of oxime therapy in case of CM ingestion is controversial (Eddleston et al. 2004; Rosman et al. 2009; Wille et al. 2013). The carbamoylated serine residue is less stable than the phosphorylated one, thereby leading to spontaneous hydrolysis and cleavage of the carbamoyl moiety. CM compounds are also used therapeutically in myastenia gravis and delirium, and paradoxically as a pretreatment for difficult-to-treat nerve agent poisoning to prevent permanent irreversible inhibition by OP by reversible CM inhibition (Inns and Marrs 1992). Hence, the identification of the compound being responsible for the cholinergic toxidrome is considered crucial. The laboratory parameter which can be determined in most routine clinical laboratories and which provides at least some information on the course of OP or CM poisoning is the butyrylcholinesterase (BChE) activity (Eddleston et al. 2008). However, BChE is not the decisive toxicological target of OP or CM and shows different kinetics with regard to inhibition and reactivation by particular OP or CM (Holmstedt 1959; Worek et al. 1999). There are high inter- and intra-individual variations of BChE activity which can be affected by a variety of parameters, e.g. drugs or nutrition (Worek et al. 2016b). In contrast, the single gene-encoded AChE is expressed on red blood cell (RBC) membranes showing comparable properties with AChE in other tissues within an individual. Despite being alternatively spliced, it is used as a surrogate parameter showing stable levels within single subjects (Massoulié et al. 1993; Soreq and Seidman 2001; Thiermann et al. 2010). To translate these findings into clinical practice, the concept of the so-called cholinesterase status was developed and consists of four independent parameters (Thiermann et al. 1997): (1) the AChE activity in a diluted patient whole blood sample, (2) the BChE activity in patient plasma samples, (3) the reactivatability of OP-inhibited AChE in a diluted patient blood sample after incubation with a supra-therapeutic concentration of an oxime and (4) the inhibitory activity of patient plasma towards uninhibited control AChE (Fig. 1).

Repeated measurement of these parameters allows addressing important questions occurring in the treatment of poisoning with AChE inhibitors (e.g. rationale of oxime administration, duration of oxime administration, persistence of AChE inhibitor in the patient) and, together with current analytical methods, to generate a comprehensive dataset which corroborates important clinical decisions (Thiermann et al. 2007; Eyer et al. 2009; Worek et al. 2016a).

## Materials and methods

### Material

Formic acid (≥ 98%), sodium hydroxide (> 99%) and hydrochloric acid (37%) were obtained from Carl Roth (Karlsruhe, Germany). Deuterated atropine (d3-atropine) was obtained from CDN Isotopes (Pointe-Claire, Quebec, Canada) and diethyl phosphate (DEP) (99.5%) from Chem Services (West Chester, UK). Sodium dihydrogenphosphate hydrate (99%) was from Fluka (Buchs, Switzerland) and n-butyl phosphonic acid (96.5%) from Lancaster (Newgate, UK). Parathion (> 99%), paraoxon (> 95%) and *p*-nitrophenol (> 99%) were obtained from LGC Group (Wesel, Germany). Acetonitrile (ACN, gradient grade), obidoxime dichloride (obidoxime), phosphoric acid (guaranteed reagent), trichloroacetic acid (TCA, guaranteed reagent), diethyl ether (Uvasol) and water (HPLC grade) were delivered by Merck (Darmstadt, Germany). Methanol and 2-propanol (both LC–MS grade) were from Riedel de Haën (Seelze, Germany). Pyridine-4-aldoxime (4-PAO), sodium 1-octanesulfonate (98–99.6%), potassium fluoride (> 99.5%), pentafluoropyridine (PFP), diisopropyl fluorophosphate (DFP), diethyl thiophosphate (DETP) (potassium salt, 98%) and atropine (free base, ≥ 98%) for HPLC–MS/MS analysis were obtained from Sigma-Aldrich (Taufkirchen, Germany). Individual human serum and plasma were delivered by Sonnen-Gesundheitszentrum (Munich, Germany). Ammonium formate (HIPerSolv Chromanorm for LC–MS) was from VWR (Ismaning, Germany) and diethyl fluorophosphate (DEFP) was synthesized by TNO (Rijswijk, Netherlands). Patient medication: Acylaminopenicillin + *β*-lactamase inhibitor (piperacillin/tazobactam) was delivered by Fresenius Kabi AG (Bad Homburg vor der Höhe, Germany), atropine by Dr. Franz Köhler Chemie GmbH (Bensheim, Germany), esketamine by Pfizer Pharma PFE GmbH (Berlin, Germany), fresh frozen plasma (FFP) was provided by the blood bank of the Klinikum Landshut gGmbH (Landshut, Germany) and obidoxime by Merck (Darmstadt, Germany).

### Analytical methods

The concept of the cholinesterase status was performed as previously described (Thiermann et al. 1997; Worek et al. 2016a). The spectrophotometer (UV/VIS Cary 300) was obtained from Agilent Technologies (Santa Clara, California, USA). AChE activity was determined according to a modified Ellman method (Worek et al. 1999). The current AChE and BChE activity, the AChE reactivatability, and the presence of inhibitory material in the patient plasma were assessed. Among the aforementioned parameters, four independent analyses are: (1) the in vivo activity of patient´s erythrocyte–AChE; (2) the reactivatability (incubation of diluted patient blood at 37 °C for 30 min with a supratherapeutic oxime concentration of 100 µM obidoxime for a defined interval to achieve a maximum possible AChE reactivation by treatment, followed by determination of AChE activity); (3) the in vivo activity of patient plasma BChE activity and IV) the inhibitory activity of patient plasma (incubation of patient plasma at 37 °C for 60 min with AChE control, followed by determination of AChE activity) (Thiermann et al. 1997; Worek et al. 2016b).

Atropine was quantified by liquid chromatography-electrospray ionization–tandem-mass spectrometry (LC-ESI–MS/MS) in a multiple-reaction monitoring mode (MRM) based on a method described before (John et al. 2010). In brief, human plasma was precipitated with acetonitrile and centrifuged. The supernatant was diluted with d3-atropine solution, which was used as an internal standard prior to injection for analysis. Atropine was quantified by external calibration using a 4000 QTRAP triple quadrupole mass spectrometer (AB Sciex, Darmstadt, Germany) coupled to an Ultimate 3000 HPLC system (Thermo Fisher Scientific, Dreieich, Germany) online.

Obidoxime quantification was performed by a method described before (Worek et al. 2010) that involved applying reversed-phase ion-pairing chromatography coupled to diode-array detection (RPIPC-DAD). In brief, human plasma was mixed with TCA for protein precipitation. The supernatant was mixed with 4-PAO, used as internal standard and diluted prior to RPIPC-DAD analysis. Quantification was carried out by an external calibration using an Ultimate 3000 HPLC system (Thermo Fisher Scientific). Reported concentrations of obidoxime refer to the pure oxime, not considering the two chloride counter ions.

Fluoride-induced reactivation of inhibited BChE was performed as published previously (Koller et al. 2010). The method was modified for making use of gas chromatography-electron ionization–tandem-mass spectrometry (GC-CI–MS/MS) instead of gas chromatography-electron ionization–mass spectrometry (GC-EI–MS). In case of diethoxy pesticides, the product of fluoride-induced reactivation is diethyl fluorophosphate (DEFP) which was detected by the following transitions: m/z 157 m/z 129, m/z 101, and m/z 129 m/z 101. Analyses were carried out by a 7890 B gas chromatograph and a 7000 triple quadrupole mass spectrometer, both from Agilent Technologies (Waldbronn, Germany). The GC system was equipped with a cold injection system (CIS 4) from Gerstel (Mülheim a. d. Ruhr, Germany).

Pesticide-hydrolysis products DEP and DETP were screened in the patient´s urine by modifying the gradient program of the LC-ESI–MS/MS method, developed for the hydrolysis products of nerve agents (Koller et al. 2010). The following transitions were monitored for DEP: m/z 152.8 m/z 124.8, m/z 78.8 and for DETP: m/z 168.8 m/z 140.8, m/z 94.9. The LC-ESI–MS/MS system consisted of an API 5000 mass spectrometer from AB Sciex, operating in the negative ionization mode, and was connected to an LC system series 200 (binary pump, autosampler, column oven and degasser) from Agilent Technologies.

The screening for unknown pesticides required 500 µl of the fluid to be screened, which was extracted twice by 500 µl diethyl ether each for 15 min. The organic layers were carefully removed and combined. Five microliter was injected into the GC-EI–MS system operating in the scan mode from m/z 50–m/z 500 using a linear temperature gradient rising from 50 to 280 °C. The GC-EI–MS system used for the screening consisted of a GC 7890 B system connected to a mass spectrometer 5977, also from Agilent Technologies, and also equipped with a CIS 4 from Gerstel. Mass spectra were compared with those of the NIST library Mass Spectral Search Program version 2.3, 2017 (Gaithersburg, Maryland, USA).

For the determination of the hydrolysis product *p*-nitrophenol that is specific for parathion and paraoxon, 1 ml of patient’s urine was mixed with ß-glucuronidase from *Helix pomatia* (Sigma-Aldrich) and phosphate buffer according to the manufacturer's recommendations. The mixture was incubated at 37 °C for 16 h followed by centrifugation at 13,440 × *g* (15,000 rpm). The supernatant was then diluted with 2.45 ml purified water and adjusted to pH 14 by 800 µl of 6-M NaOH to ensure phenolate formation. This alkaline solution was introduced to an anion exchanger resin (Oasis Maxx, 30 mg, 3 ml, Waters, Eschborn, Germany) which was preconditioned by 2.5 ml acetone, 2.5 ml purified water and finally by 2.5 ml of 0.1 M NaOH. After adsorption was complete, the bed was rinsed with 2.5 ml acetone and rigorously dried under full vacuum for 15 min. For derivatization, 1.1 ml of a solution of 200 µl PFP in 1.1 ml of hexane was applied and allowed to sink into the bed by gravity (Kojima et al. 2003, 2004). After 10 min of incubation, the stopcock was opened to let excess PFP flow out. For elution, the bed was rinsed three times with 1 ml hexane collecting all of the eluates. The combined hexane-eluates were then concentrated to about 400 µl in a centrifugal evaporator (RVC, Christ, Osterrode, Germany). 1 µl of the residue was then splitless-injected into the GC-time-of-flight (TOF)-MS-System which consisted of a GC 7890 B from Agilent Technologies equipped with a CIS 4, and a Multiple Purpose Sampler (MPS) from Gerstel and of a TOF mass-selective-detector (MSD) Pegasus 4D from Leco (Mönchengladbach, Germany). The CIS was started at 50 °C with a hold time of 0.1 min and then heated to 260 °C (1 min hold). The oven temperature also started at 50 °C (1.2 min hold) and was raised to 300 °C (5 min hold) at a rate of 10 °C/min. MS detection (scan mode) was programmed from 50 to 500 m/z.

## Results

### Case report: clinical signs and intensive care treatment

An 83-year-old patient was reported to be deeply unconscious at home with a Glasgow Coma Scale of 3. On arrival of the emergency team, the patient presented with bradycardia (30 bpm), hypotension (systolic blood pressure (BP) 60 mmHg) and aspiration. Furthermore, a blue-stained vomitus covering the patient’s mouth and upper airways emitted a pungent, garlic-like smell. After anesthesia with fentanyl and propofol, immediate intubation and mechanical ventilation were performed for the airway protection and to avoid aspiration. Atropine and epinephrine were applied to counteract bradycardia and hypotension. At the hospital, activated charcoal was administered by gastroscopy after gastric lavage. Routine laboratory analysis showed acute renal failure (creatinine 1.7 mg/dl), lactic acidosis and an almost complete inhibition of plasma BChE determined with an automated clinical routine analyzer (< 200 U/l, reference: 5320–12,920 U/l). The typical signs of severe cholinergic crisis including miosis, salivation, bradycardia (50 bpm) and hypotension (BP 70/30 mmHg) occurred 12 h post-admission, the delay being probably caused by the initial treatment with vasoconstrictors. Due to a suspected OP poisoning, an antidotal therapy comprising atropine and obidoxime was initiated at day 0: a bolus of obidoxime (250 mg) followed by a continuous infusion of 750 mg/day. To initiate additional degradation of OP within the body, the patient received a total of 10 units of FFP (4–3–2–1) on 4 consecutive days (day 1–4) according to Dayananda et al. (2016); for more details on the administration of FFP in OP poisoning, the reader is referred to Wille et al. (2014). Atropine was administered until the clinical signs ceased (absence of miosis and salivation, dry axilla, clear lung, heart rate > 80 bpm). Nevertheless, the dosage of atropine was challenging. This became clearly evident at day 4 when continuous atropine dosages of 1.0 mg/h antagonized the muscarinic signs, but resulted in brief episodes of supraventricular tachycardia (up to 160 bpm).

In addition, esketamine was administered for neuroprotection at a dosage of 1277.5 ± 595.2 mg/day, starting at day 2 for a total of 10 days.

**Fig. 2:**
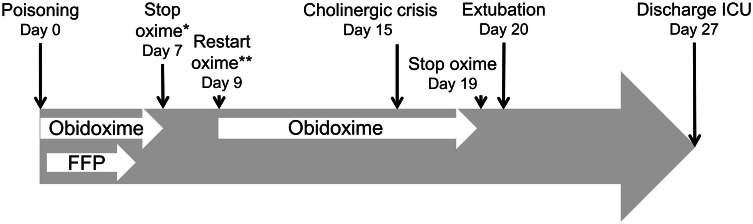
Timeline displaying key events in the current case report. At day 7 (*), the oxime therapy was stopped due to suspected pirimicarb poisoning and on day 9 (**), the therapy was restarted due to laboratory confirmation of OP poisoning

On day 7 after admission, the police informed the ICU that a hand-labeled “pirimicarb” bottle was found in the shed of the poisoned patient. Pirimicarb is a CM pesticide and, thus, CM poisoning was suggested instead of OP poisoning. Due to the new tentative diagnosis, owing to carbamate poisoning and its arising complications (aspiration pneumonia, acute pancreatitis and a subileus), obidoxime therapy was discontinued at day 7 as summarized in Fig. 2.

**Fig. 3:**
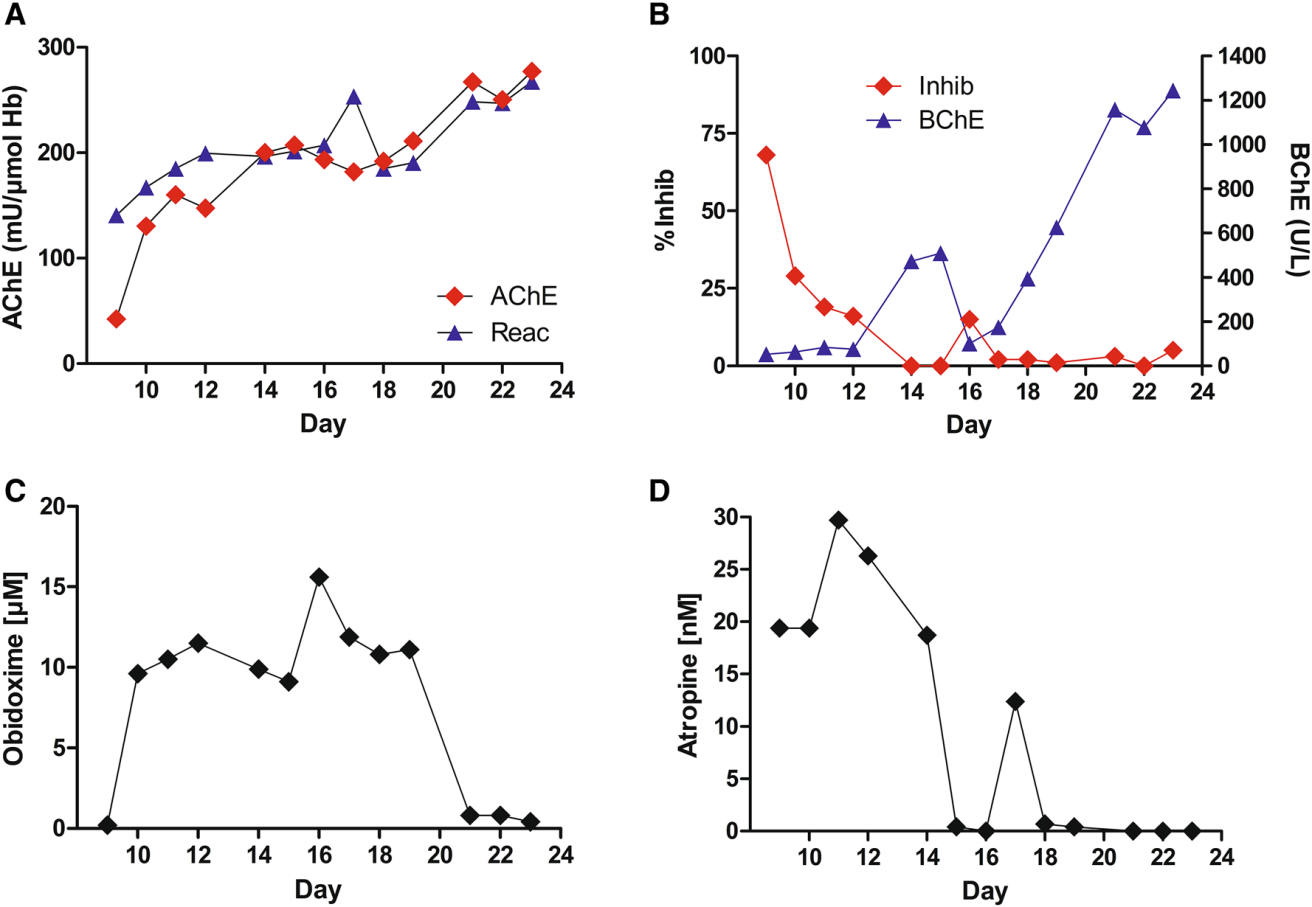
ChE status and antidote concentrations of the patient receiving atropine and obidoxime. The AChE in vivo activity (AChE) and maximal possible AChE activity after incubation with 100 µM obidoxime ex vivo is given in mU/µmol Hb on different days after admission (Reac, **a**). Inhibitory activity is given in % inhibition of lyophilized-test erythrocyte**–**AChE (Inhib) and BChE activity in U/l (**b**). Obidoxime (**c**) and atropine (**d**) plasma concentrations are given in µM or nM, respectively. The recommended dose of 750 mg obidoxime was administered by daily continuous infusion and resulted in therapeutic concentrations of ~ 10 µM (**c**). Atropine was administered by bolus injection, depending on the severity of cholinergic signs with a total amount of 300 mg within 15 days of treatment (**d**)

As BChE remained inhibited and the clinical conditions did not improve, the Bundeswehr Institute of Pharmacology and Toxicology was consulted on day 9. To get more detailed information on the course of poisoning, analysis of the cholinesterase status was started (Fig. 1). This analysis suggested OP poisoning, in contrast to the carbamate found by the police, and indicated a potential benefit from an obidoxime therapy. Hence, the latter was restarted on day 9, accompanied by a low-dosage atropine therapy of 0.5–1.0 mg/h. Due to the high level of inhibitory activity (~ 20% inhibition of AChE activity by the patient´s plasma until day 11, Fig. 3b), cholinergic signs persisted until day 15. Consequently, the weaning from mechanical ventilation was prolonged. A brief, but severe reoccurring cholinergic crisis on day 15 deteriorated the weaning process. Furthermore, aspiration pneumonia that required treatment with antibiotics (acylaminopenicillin + *β*-lactamase inhibitor) contributed to the necessity of mechanical ventilation until day 20. The patient could be discharged from the ICU after 27 days and full recovery was reported.

The concept of the cholinesterase status and analytical chemistry related to clinical signs of the poisoned patient

Due to the delayed start of extensive laboratory monitoring, only samples from day 9 to day 23 could be analyzed with the cholinesterase status (Fig. 3a, b).

**Fig. 4:**
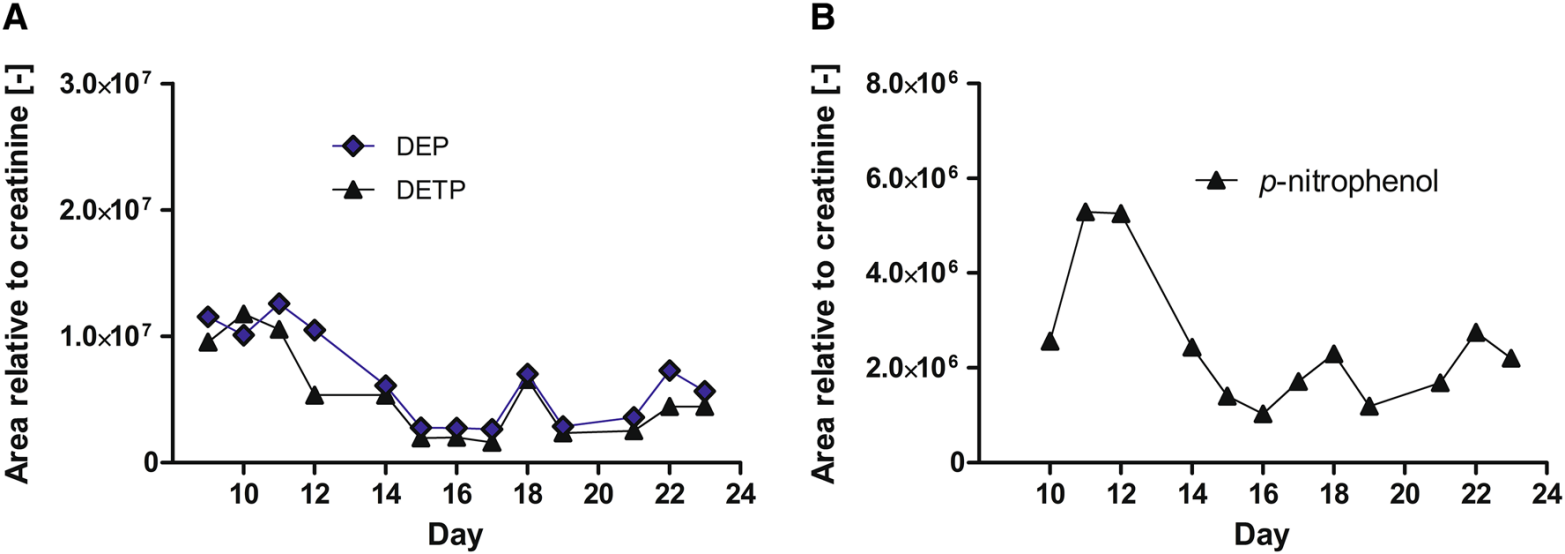
Time course of the alkyl-phosphate biotransformation products of parathion, diethyl thiophosphate (DETP) (**a**) and paraoxon, diethyl phosphate (DEP) (**a**) and the leaving group of parathion/paraoxon, *p*-nitrophenol (**b**) in patient urine. The acute cholinergic crisis on day 15 led to a delayed increase of DEP and DETP on day 18 (**a**) and to similar kinetics of *p*-nitrophenol (**b**)

AChE activity in patient samples was low on day 9 but increased promptly during obidoxime treatment from 42 to 130 mU/µmol Hb, and then more slowly to about 200 mU/µmol Hb until day 14 (Fig. 3a). This is in line with the decrease of inhibitory activity from day 9–12 and subsequent increase of newly synthesized BChE, suggesting an absence of poison in the body of the patient (Fig. 3b). On day 15, the patient re-developed a severe, but brief cholinergic episode that necessitated the administration of high dosages of atropine (48 mg/24 h), thereby resulting in an atropine-plasma level peak of 12 nM on day 17 (Fig. 3c). This acute clinical deterioration was matched by a > 80% drop in BChE activity (from 510 U/l on day 15–100 U/l on day 16) and an increase of inhibitory activity (from 0% on day 15–15% on day 16), and a dip of AChE activity (Fig. 3a, b). Further analysis via the method of fluoride-induced reactivation of inhibited BChE via GC-CI–MS/MS proved diethoxy pesticide to be present in the patient’s body. Besides, a pesticide-biotransformation product screening via LC-ESI–MS/MS found the alkyl-phosphate biotransformation products of parathion, DETP and paraoxon, DEP, and the nitrophenolic biotransformation product of parathion and paraoxon, *p*-nitrophenol (Fig. 4).

After the re-onset of cholinergic crisis on day 15, the clinical condition improved, no further atropine had to be administered and the level of RBC–AChE and BChE activity continuously increased. In total, 300 mg of atropine and 10 g of obidoxime were administered and the patient was discharged from ICU on day 27.

## Discussion

Despite extensive experience and the best critical-care treatment, mortality of OP and CM poisoning is still high (Eddleston et al. 2004; Hrabetz et al. 2013; Mew et al. 2017). In the past years, several attempts have been suggested for further optimization of the therapeutic strategy. One option consists in the determination of the time frame in which the application of oximes is beneficial (Worek et al. 2016a). Hence, identifying patients in whom oximes will be able to reactivate OP-inhibited AChE and might, thereby, improve the clinical condition. Furthermore, differentiating OP from CM poisoning as a possible cause for a cholinergic crisis is important, and there is an ongoing debate on potential harmful effects of oximes in CM poisoning (Eddleston et al. 2004; Rosman et al. 2009; Wille et al. 2013).

In the present case, the agent responsible for the cholinergic crisis was not known initially, which is representative of many cases of self-poisoning. However, the most likely cause for the persistent toxidrome of miosis, bradycardia and salivation was OP poisoning (Eddleston et al. 2004). Hence, the competitive antagonist of ACh at the muscarinic receptor, atropine, was administered until full atropinization was achieved (systolic blood pressure > 80 mm Hg, heart rate > 80 bpm, and drying of pulmonary secretions) (Eddleston et al. 2008; Hulse et al. 2019). Obidoxime was applied according to the standard recommendation of a bolus of 250 mg, followed by the continuous infusion of 750 mg/d. This resulted in plasma concentrations of about 10–15 µM (Fig. 3c), which are generally deemed effective and safe (Thiermann et al. 2002; Eyer et al. 2009). Due to the latter confirmed, high poison load, it may be assumed that no relevant net reactivation occurred in the beginning. This was substantiated by the persisting cholinergic signs calling for high atropine dosages over several days (with a peak dose of 64 mg on day 3). Moreover, the clinical situation declined due to aspiration pneumonia (despite an early intubation), which is encountered in up to 30% of all OP-poisoned patients (Hulse et al. 2014). The severe, life-threatening clinical condition led to the additional administration of 10 units FFP on 4 consecutive days (day 1–4), to facilitate various catalytic and stoichiometric detoxification mechanisms of OP, including pseudocatalytic scavenging (Nachon et al. 2013; Wille et al. 2014). This concept comprises the administration of exogenous cholinesterases and an oxime to increase the 1:1 stoichiometry of enzyme detoxification. Recently, some studies suggested a potential benefit of human FFP in OP poisoning (Güven et al. 2004; Pichamuthu et al. 2010; Dayananda et al. 2016). However, these measures did not improve the clinical situation of the current patient. A possible explanation might be the latter-identified-OP parathion and its active biotransformation product, paraoxon, respectively. The reactivation of paraoxon-inhibited BChE with obidoxime is characterized by a low-reactivity constant (*k*_r_ 0.07 min^−1^) and a low-affinity constant (*K*_D_ 317 µM) (Horn et al. 2015). On the basis of these kinetic parameters and the in vivo obidoxime concentration (10 µM), a reactivation half-time of paraoxon-inhibited BChE of > 300 min was calculated, being much too low for effective catalytic scavenging.

On day 7, a half-empty bottle labeled with the name “primicarb” was reported to be found at the scene by the police suggesting CM poisoning instead of OP, which caused a change in the therapeutic strategy (Fig. 2). As CM–AChE conjugates show a high rate of spontaneous reactivation (Eddleston et al. 2004) and due to the assumption that oximes provide no benefit in carbamate poisoning (Rosman et al. 2009; Wille et al. 2013), it was decided to stop obidoxime treatment. GC–MS analysis later revealed a high amount of pirimicarb in the pirimicarb-labeled flask. This indicates that evidence found at the scene might be misleading and trigger inappropriate therapeutic measures (stop of the obidoxime therapy). Hence, all laboratory attempts facilitating early diagnosis are important.

In this critical situation with contradicting diagnoses, the repeated measurement of the cholinesterase status allowed a better insight into the course of poisoning. It contributed to clinical decisions concerning the continuation of oxime therapy. The results on day 9 showed that AChE activity in patient samples was very low, but could be successfully reactivated with obidoxime (Reac, Fig. 3a) despite moderate inhibitory activity (Inhib), while BChE remained almost completely inhibited (Fig. 3b). This was due to the fact that the reactivation constant *k*_r2_ of obidoxime-induced reactivation of paraoxon-inhibited AChE is 20 mM^−1^ min^−1^, whereas that of paraoxon-inhibited BChE is only 0.23 mM^−1^ min^−1^ and, thus, ~ 100-fold lower compared to the *k*_r2_ of AChE (cf. Fig. 3a, b) (Worek et al. 2012; Horn et al. 2015). Relying only on BChE data, one could get the misleading impression that obidoxime administration does not result in reactivation and, therefore, not lead to the improvement of synaptic AChE activity and, a better neuromuscular transmission consequently (Thiermann et al. 2005).

The results from the concept of the cholinesterase status prompted the reconsideration of the tentative diagnosis of CM poisoning, since only OP–AChE conjugates can be reactivated by oximes, whereas it does not interfere with carbamoylated AChE (Wille et al. 2013). As expected in OP poisoning, obidoxime concentrations of about 10–15 µM were able to increase RBC–AChE activity within a short time (Thiermann et al. 2002; Eyer et al. 2009) (Fig. 3a). However, the remaining inhibitory activity was still high enough to prevent complete reactivation until day 14. After the re-start of the obidoxime infusion on day 9, reactivatability was at a level of ~ 200 mU/µmol Hb. This level is clearly below the normal range of 531–796 mU/µmol Hb and indicates aging (Worek et al. 2016b). The aging half-life of diethyl-phosphorylated AChE is about 30 h in the absence of poison and oximes (Worek et al. 1997). Previous studies demonstrated a doubled aging half-life in parathion-poisoned patients by continuous obidoxime administration (Thiermann et al. 1997, 1999), probably being a result of continuous cycles of reactivation and re-inhibition. It may be assumed that the patient benefited from obidoxime therapy by maintaining a partial reactivatability of AChE. Nevertheless, a recent review indicated that an oxime therapy should be started in the absence of exact knowledge, whether an OP or CM is involved and maintained, until the identification of the agent responsible for the cholinergic crisis (Rosman et al. 2009). A possible substantial benefit in OP poisoning justifies an oxime therapy, even though it might be of little benefit in CM poisoning.

A pesticide-biotransformation product screening via LC-ESI–MS/MS identified the alkyl-phosphate biotransformation product of parathion, i.e. DETP and paraoxon, i.e. DEP and the leaving group of parathion/paraoxon, *p*-nitrophenol (Fig. 4). DETP and *p*-nitrophenol were rapidly eliminated into the urine and allowed a thorough monitoring of parathion exposure (Morgan et al. 1977). In the current case, the brief cholinergic crisis at day 15 resulted in an increase in these biotransformation products and suggested an acute parathion challenge (Fig. 4). This cholinergic crisis on day 15 requires thorough consideration. The decrease in BChE activity and the increase in the inhibitory potential (Fig. 3b) suggested an acute challenge with parathion and its active biotransformation product paraoxon, respectively. In the current case, the anti-muscarinic therapy with atropine led to intestinal paralysis which has been described as a serious side effect of atropine therapy (Beards et al. 1994). Necessary laxative measures on day 13 and 14, to treat this condition, might have led to the redistribution of parathion from the bowel. Hence, the possibility of re-occurring cholinergic crisis after prolonged anti-muscarinic therapy has to be taken into account. Fortunately, this event lasted only for a short time and cholinergic crisis subsided fast.

In conclusion, the concept of the cholinesterase status and the measured enzyme activities assisted in the diagnosis and the management of the cholinesterase-specific part of OP poisoning. Besides, the distinction from the initially suspected CM poisoning facilitated therapeutic decisions, e.g. the administration and the duration of oxime application.
